# Characterization of the Types of Sweeteners Consumed in Honduras

**DOI:** 10.3390/nu10030338

**Published:** 2018-03-11

**Authors:** Adriana Hernández, Adriana Beatriz Di Iorio, Jeffrey Lansdale, María Belén Salazar

**Affiliations:** 1 Department of Food Sciences, Zamorano University, 11101 Tegucigalpa, Honduras; adi@zamorano.edu (A.B.D.I.); salazarmb27@gmail.com (M.B.S.); 2 President’s Office, Zamorano University, 11101 Tegucigalpa, Honduras; jlansdale@zamorano.edu

**Keywords:** soft drinks, nutritional labeling, corn syrup, nutrient profile

## Abstract

Sweeteners are found in all types of foods, and their high consumption is associated with chronic degenerative diseases, such as diabetes and obesity, among others. A characterization was carried out of food products with sweeteners from the three biggest supermarkets at a national level; they were identified by the list of ingredients and classified according to caloric or non-caloric intake, and pursuant to their country of origin. A statistical interpretation of results was made using descriptive measures such as the number of times the sweeteners were found in the formulation of the products and how many of them were found in a product at the same time. In total, 341 products were evaluated and classified according to the processed food categories of the Pan American Health Organization (PAHO) nutrient profile. The category of beverages had the highest quantity of products with sweeteners, and their consumption by the inhabitants represents a high exposure. Overall, 60.1% of the products evaluated were of US origin; these US exports have a significant impact on the Honduran market. A high-fructose corn syrup caloric sweetener was the one most frequently found in these products; at least 51% are combined with additional sweeteners to increase the sweetening effect.

## 1. Introduction

The presence and impact of artificial sweeteners in Latin America are of growing concern, particularly from a perspective of basic human health and nutrition. While this article is a case study of trends in Honduras, the findings could represent general tendencies in all countries in Latin America and the Caribbean. To better understand how non-nutritive sweeteners have been assimilated in the common diet of Honduran society over the past twenty or more years, it is helpful to consider contextual factors.

Honduras is a low middle-income country that faces major challenges, with more than 66% of the population living in poverty in 2016, according to official data. In rural areas, approximately one out of five Hondurans lives in extreme poverty, or on less than US $1.90 per day [1].

The economy of Honduras is based on agricultural production, and particularly coffee, one of its main export items. Steadily rising production in palm oil and fruits, as well as robust yields of food crops intended primarily for the domestic market, have also contributed to the growth of the agricultural sector [2]. The food and beverage industry, which accounts for about 45% of total manufacturing, grew by just 2.3%. This type of industry uses high fructose corn syrup for its great sweetening power and its low costs, whose high consumption generates metabolic complications.

Processed foods are imported for sale in different types of establishments (supermarkets and local stores) nationwide, and those ready to eat mainly in urban areas [3], with little health regulation regarding the convenience of the ingredients and of nutritional labeling, more oriented to the microbiological quality and contaminants [4]. In particular, the issue of sweeteners has not been regulated by government (consumer protection/economy and health).

The quantity and type of sweeteners in the products according to their origin depends on the legislation in the country of origin. The United States Food and Drug Administration (FDA) sets limits for products in the United States according to the consumer’s body weight, establishing upper limits for daily intake (Table 1), which serve as a guide according to their consumption and presence in food. However, by not declaring the quantity, it is difficult to estimate their consumption in conjunction with the different foods that contain them.

The last food consumption study carried out in Honduras was in the 1980s, with only some information on apparent consumption from the “2004 Living Conditions Survey” [6] where the high consumption of soft drinks is already observed (73% of the non-poor population and 37% poor) as well as other processed foods. In addition to this information, there are only studies in local populations [7] that show that these types of products are among the most frequent.

The replacement of sugar by non-caloric sweeteners has occurred in a short time in the processed foods, without knowing the type or amount used and less the amount of consumption by people who ingest different processed foods in their daily diet, which could be dangerous to their health. It is difficult to do justice to the broad ranging implications of these demographic and cultural factors in an analysis of the impact of new dietary substances in a country like Honduras. There have been drastic changes in the past 30 to 40 years in the kind of foods being consumed in Honduras. Decades ago, a typical breakfast (desayuno tipico), even in rural areas, consisted of beans, eggs, tortillas, cheese, sour cream, avocado, coffee, and occasionally a piece of meat.

For people who are poorly educated [8], it is difficult to understand that something that tastes as good as a soda might be harmful to one’s health. Governments with limited resources cannot afford communication campaigns to warn their citizens of the health hazards of consuming sweetened substances.

The need for additional research into the marketing, availability, consumption, and impact of sweetened foods in poor countries such as Honduras is imperative. This is an initial study of the presence of artificial sweeteners in a Latin American context. In Honduras, there is no previous information published in this regard. The purpose of this study is to analyze the information available about food for general consumption to characterize the types of sweeteners consumed in Honduras.

## 2. Materials and Methods

A preliminary study was carried out in Tegucigalpa based on the review of the labels of processed foods with sweeteners, according to the nutrient profile model of the Pan American Health Organization (PAHO) as follows: soft drinks/juices/nectars, sweets, salty snacks, cereal bars, breakfast cereals, sugary dairy products, sausages and others that includes sauces, syrups, creams, jams, jellies, coffee and other items, such as chewing gum [9].

A list of supermarkets with national presence (supermarket chains) was prepared and of these the three largest were selected and visited by trained personnel to review the different shelves with Processed foods available. The only requirement for the products sampled was that the list of ingredients indicated the presence of at least one non-caloric sweetener. The goal was to identify 250 products but a total of 341 were identified. The product containers were photographed. An exhaustive conventional and dietetic product search was carried out.

Subsequently, a database was created with the name of the product, producing company, country of origin, nutritional labeling and list of ingredients. Information was collected from February to September 2017. The generation of a database in Excel (Microsoft, Redmond, WA, USA) and statistical interpretation of results using descriptive measures, specifically frequency distribution tables, was carried out in the Human Nutrition Laboratory at Zamorano University in Honduras.

The information was organized according to the quantity of products with sweeteners by category, to identify those with a greater presence; the country of origin of the products; the origin of the sweeteners (caloric or non-caloric); the number of times the sweetener was found in each food; and the number of combined sweeteners present in the food.

The parameters shown in Table 1 were used to classify the origin of the sweeteners (caloric or non-caloric).

## 3. Results

### 3.1. Sweeteners by Food Category

The Honduran population has a high exposure to caloric or non-caloric sweeteners (Table 2) present in all food categories, without apparent national regulation. With regard to beverages, they dominate in the categories of evaluated processed foods, showing the greatest number of products with sweeteners in their formulation, pursuant to the list of ingredients. After beverages, the categories of foods with the highest sweetener content included sweets, salty snacks, cereal bars and breakfast cereals (Table 3). In the “other” category, sauces and syrups top the list of products containing sweeteners. The category denominated “others” has a sub-category of “several,” which includes chewing gums, baking mixes and fruit preserves.

### 3.2. Origen of Processed Foods with Sweeteners

**Figure 1:**
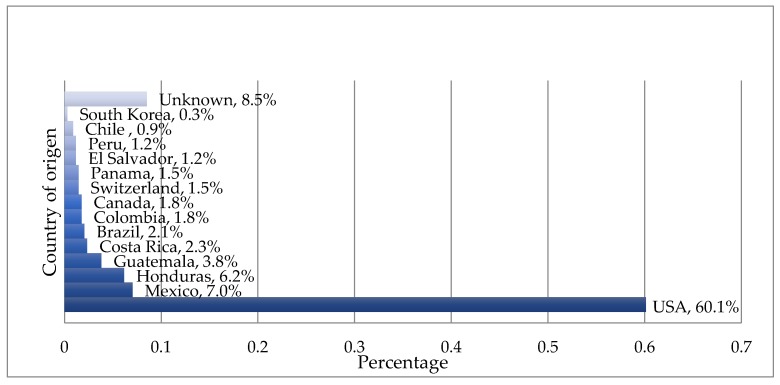
Classification of products by country of origin.

The imports and greatest number of the products evaluated come from the United States; this is facilitated by the free trade agreement it has with Central America and the Dominican Republic. With respect to Latin America, products from 10 countries were identified, including national products. In 8.5% of the products, it was impossible to identify the producing country (Figure 1). In addition, other evaluated products were imported from the following countries: Canada, 1.8%; Switzerland, 1.5%; and South Korea, 0.3%. Apart from the US, Mexico is the second country from which Honduras imports this type of products with 7%, which is close to the national contribution (6.2%).

### 3.3. Number of Times That Sweeteners were Mentioned in Processed Foods and Frequency in the Content of the Same Product

To differentiate the possible effects and/or benefits they may have on human health, the sweeteners found in the products were classified according to their caloric content (Table 4). Four non-caloric and twelve caloric sweeteners were identified in the total product evaluation. Overall, 95% and 24% of the products contain caloric and non-caloric sweeteners, respectively, which show the trend of their availability and consumption.

**Figure 2:**
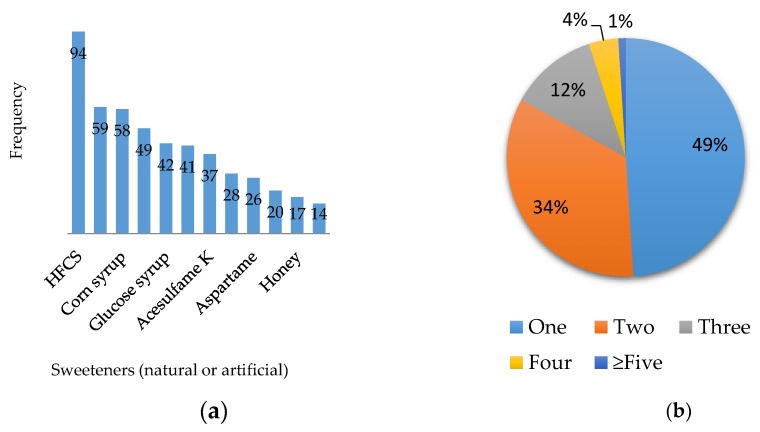
(**a**) Number of times the sweeteners were found; and (**b**) frequency of sweeteners present in the products (one = only one sweetener; two = until two sweeteners; three = until three sweeteners; four = until four sweeteners; five or more = five or more sweeteners present in the food product.

The sweeteners were grouped according to the frequency in which they were mentioned on the labels of the products evaluated as shown in Figure 2. High-fructose corn syrup (HFCS) was found in the greatest presence, possibly due to its low costs [11]; it was found especially in the category of drinks and syrups, as well as in some cookies and bars that included fruits or fruit flavors in their formulation. HFCS was followed by sucralose and corn syrup with 35 and 34 fewer percentage points, respectively. Sucrose is present in 14% of the products and honey in 17%, while sweeteners such as aspartame were found in 26% of the products. On the other hand, in the same figure, it can be observed that 51% of the products evaluated have more than one sweetener present in their formulation.

## 4. Discussion

The 341 food products evaluated in the study in their different categories are widely consumed by the general population at national level; some of these were mentioned in the national food pattern in the latest consumer study [6], where it is observed that beverages, dairy products, creams, jams, sausages and others are part of the daily Honduran diet; this indicates a high exposure to sweetener consumption, which is associated with chronic degenerative diseases related to nutrition, such as obesity, diabetes, cardiovascular diseases, Parkinson’s disease and Alzheimer’s disease [12,13]. The incidence and prevalence of these diseases have not been limited to countries with high purchasing power. In 2017, the World Health Organization (WHO) stated that 47.6% of the Honduran population was overweight (52% women) and that 16.3% suffered from obesity [14]. Poor eating habits and lack of basic nutritional education in developing countries lead to alarming numbers of affected individuals [15].

An epidemiological and clinical review showed that the food selection of the population at present generates obesity in epidemic proportions. The risk of diabetes, weight gain and cardiovascular disease [16,17] is associated with the fructose content [18], and more recently to the incidence of diabetes in postmenopausal women [19], its effects on hypertension, increased hepatic metabolism, Novo synthesis and lipogenesis, and the production of uric acid, leading to the accumulation of visceral and ectopic fat [20,21,22]. However, it is difficult to establish the degree to which they are related between them due to other nutritional, cultural and genetic factors that intervene. The conclusions of these studies are limited to the number of controlled trial studies that were conducted [23].

The high availability and accessibility of these drinks throughout the population, as well as the lack of taxation regulations to reduce consumption, result in the population maintaining or increasing the ingestion of non-caloric and caloric sweetened beverages. These products are targeted at the population, exposing it to a high consumption of beverages with large amount of sugar, HFCS and other sweeteners that make them part of the daily diet of Hondurans; this leads to the population preserving these patterns of consumption and developing chronic diseases in an accelerated manner and at younger ages, as evidenced by studies that there are significant changes in the proportion of visceral fat and low weight gain in pediatric populations that consumed carbonated drinks over a period of 18 months with the addition of 34 mg of sucralose and 12 mg of acesulfame potassium [24]. Even in populations diagnosed with diabetes, studies indicate that the prevalence of consumption of non-nutritive sweeteners is 96%, with the category of beverages being the second most consumed [25]. This is in line with results of the study carried out at Zamorano, which identified the category of beverages with the greatest exposure to any type of sweeteners.

Pursuant to the PAHO nutrient profile, the amount of sugar added per product must not exceed 10% of the total calories contributed per serving; these must be declared on the nutritional label based on new legislation in the United States. In turn, simple sugars must not exceed 10% of the total carbohydrates in the daily diet [9], although other instances like The American Heart Association maintain that the total consumption of sugar a day should not be more than six teaspoons for women and nine for men (17). The quantity and type of sweeteners in the products according to their origin depends on the legislation in force in the country of origin.

The United States Food and Drug Administration (FDA) sets limits for products in the United States according to the consumer’s body weight, establishing maximum daily recommendations (Table 1), which serve as a guide for their consumption and presence in food. With regard to the nutritional label, the Codex Alimentarius does not establish regulations to declare the number of added non-caloric sweeteners, nor is there national legislation that requires mentioning their quantity; therefore, there is ignorance regarding their contribution and consumption in the diet [26], which is related to indifference of having the respective regulations and preparation of proposals for the benefit of the population, regarding the prevention and control of obesity and chronic diseases despite the efforts that international organizations have made [27].

One third (34%) of the food products imported by Central American countries come from the United States, 15% from Mexico, 6% from the Netherlands and 4% from Colombia. Honduras maintains free trade with the United States, eliminating many tariffs and other restrictions for US goods to enter the Central American market [28], which is reflected in the fact that 60% of the products evaluated in this study come from North America. These imports have come to modify consumption patterns with adverse effects on health and changes in the cultural food pattern. The United States leads the problem of obesity [29] related to poor eating habits and excess of foods with a high content of sugar, salt and total, saturated and trans fats. For the same reasons, in Mexico, more than 70% of the adult population suffers from being overweight or obese [30].

According to their origin, sweeteners have the potential to cause health effects; beverages sweetened with sugar have accumulated evidence of leading to weight gain due to a reduction in the feeling of satiety relative to solid foods. On the other hand, products sweetened with artificial non-caloric sweeteners generate an imbalance in the regulation of appetite, due to discordance between intense taste of sweetness and the lack of caloric intake. Therefore, their consumption stimulates appetite and consequently the gaining of weight [25]. A greater amount of research should be carried out in this line since the results are inconclusive [31].

In attempts to reduce sugar consumption, non-caloric sweeteners have gained popularity [32], and studies such as that of Japan (2014), reports that diet soda consumption was significantly associated with an increased risk of diabetes [33]. The sweetening power of sweeteners such as stevia, which is 100 to 300 times greater than sugar [34], is very attractive to the food industry, due to its low costs and as a result of the growing demand for light products in the elaboration of beverages, sweets, jams, chewing gum, baked products, jams and yoghurts, among others [32], because less quantity is used in the formulation with the same effect as sugar. The low production costs of the raw material of HFCS, which includes corn starch, are because this crop is subject to agricultural subsidies in the United States, increasing its availability and its intake at a national level and in all the countries where the products are exported. The excessive and constant consumption of fructose progressively increases its rate of absorption and consequently the availability of Acetyl coenzyme A (acetyl-CoA), which causes an increase in lipid synthesis [32]. The consumption of fructose instead of glucose does not have significant differences in the result of insulin or blood glucose concentrations [35], which is why its consumption can be associated with the same chronic degenerative diseases caused by disorders in the secretion of different hormones such as insulin. Honduras, like the rest of Central America, has a high production of sugarcane, leading to a wider use of sucrose.

Sucralose, which was the second most commonly found sweetener, is a modified, trichlorinated form of common sugar (sucrose) without calories and 600 times sweeter. It has a flavor that differs considerably from ordinary sugar and does not decompose with heat. It is widely used throughout the world, alone or with other sweeteners, and can be found in many foods and beverages [36]. Sucralose, similar to other natural or artificial sweeteners found, is associated with high risk of obesity, diabetes, heart disease and Alzheimer’s or Parkinson’s disease [13].

These sweeteners are combined due to synergistic effects that enhance their sweetening power with the use of less quantity of product, reducing costs, as well as guiding the development of a new range of products that contributes sweetness to food without the usual calories [37]. The industry has effectively identified the pros and cons of each sweetener. For example, they state that it is necessary to know the profile of temporary sweetness, since some sweeteners have a very rapid onset (for example, fructose), while others have a slow start or last longer (for example, aspartame). If the sweetener used to replace sugar has a time profile very different to sugar, another sweetener is often added to the mixture to cope with the difference. Hence the popularity of acesulfame K, which has a rapid onset, while the sweetness of high intensity sweeteners (often aspartame or sucralose) accumulate later. In addition, aspartame and acesulfame K have a robust synergy [32]. Multiple alternatives are sought, so that caramels and chocolates offer the same taste and sweetness, but with fewer calories with the belief that they will reduce the risk of developing diseases such as diabetes or obesity [38], and without considering the need for long-term studies to confirm this.

What the industry takes as a business opportunity increases the exposure of more than one sweetener at the same time in the consumption of a single product. Within the 341 products evaluated, 1% contained more than five sweeteners declared in the list of ingredients; these were mostly products in the category of sweets and chewing gums. Likewise, the 5% of products containing four sweeteners in their formulation were mostly cookies, bars and chewing gums. Although most of these chewing gums contain non-caloric sweeteners, their health risks continue to be present because they stimulate appetite with a high sensation of sweetness without any caloric intake, which encourages the population to eat more and subsequently suffer from the eating disorders [22] mentioned above, not to mention the effects on the microbiota beyond affecting the weight of people [39].

The lack of regulations regarding caloric and non-caloric sweeteners results in their indiscriminate use, leading the population to suffer chronic degenerative diseases and other social problems that cause obesity. The need for nutritional education, accurate regulation and research in this line is important for making significant integral progress regarding the prevention and control of obesity and related chronic diseases.

## 5. Conclusions

Globalization leads to a sharing of diet patterns and other cultural elements, enabling the incidence and prevalence of obesity or diabetes to transcend borders. Sweeteners, both caloric and non-caloric, are present in many food products available in the Honduran market, resulting in a broad consumption by the population. Especially in Honduras, deficiencies in nutritional education, lack of regulation on the use of sweeteners, and limited information on their health consequences favors their excessive consumption.

The information collected represents a basis for future research on the presence, use and consumption of sweeteners in the Honduran diet, as well as on their relationship with chronic degenerative disease rates in inhabitants. In addition, the information will contribute to the creation of educational solutions to help consumers select healthier foods.

## Figures and Tables

**Table 1:** Acceptable daily intake of non-caloric sweeteners approved by the United States Food and Drug Administration (FDA).

Sweetener	Acceptable Daily Intake by the FDA (mg/kg)
Acesulfame potassium (Acesulfame K)	15
Advantame	32.8
Aspartame	50
Neotame	0.3
Saccharin	15
Sucralose	5
Steviol glucosides	4

Source: (FDA 2016) [5].

**Table 2:** Classification of sweeteners according to their caloric intake.

Origin	Classification	Sweetener
Caloric	Sugars	Sucrose, glucose, dextrose, fructose, lactose, maltose, galactose, isomaltulose, trehalose, tagatose, sucromalat
	Natural	Honey, cream syrup, palm or coconut sugar, sorghum syrup
	Modified sugars	High fructose corn syrup, caramel, inverted sugar
Non-caloric	Sugar alcohols	Sorbitol, xylitol, mannitol, erythritol, malititol, lactitol, glycerol
	Artificial	Aspartame, sucralose, saccharin, neotame, acesulfame K, cyclamate, neohesperidin dihydrochalcone (neohesperidine DC), alitame, advantame
	Natural	Stevioside

Source: Torresani, M.E., et al. [10].

**Table 3:** Quantity of products with sweeteners classified by food category.

Category of Products	Number of Products with Sweetener
Soft drinks/juices/nectars	81
Sweets	62
Salty snacks	59
Cereal bars	23
Breakfast cereals	23
Sweetened dairy products	10
Sausages	6
Others:	22
Sauces	19
Syrups	11
Several (gum)	10
Creams	9
Jams	4
Jellies	2
Total	341

Source: own elaboration.

**Table 4:** Classification of sweeteners present in food according to their caloric content.

Non-Caloric	Caloric
Acesulfame K	High-fructose corn syrup (HFCS)
	Glucose
Aspartame	Glucose syrup
Stevia	Maltodextrin
Sucralose	Fructose
	Honey
	Sucrose
	Lactose
	Mannitol
	Sorbitol
	Corn syrup
	Inverted sugar
